# A Fluorescent Immunochromatography Test Strip for the Rapid Identification of SVV and FMDV

**DOI:** 10.1155/2024/1628008

**Published:** 2024-08-12

**Authors:** Liuyue Yang, Chengfei Li, Xinghua Chen, Kun Li, Zengjun Lu, Xiangmin Li, Meilin Jin, Ping Qian

**Affiliations:** ^1^ National Key Laboratory of Agricultural Microbiology Hubei Hongshan Laboratory Huazhong Agricultural University, Wuhan 430070, Hubei, China; ^2^ Laboratory of Animal Virology College of Veterinary Medicine Huazhong Agricultural University, Wuhan 430070, Hubei, China; ^3^ Key Laboratory of Preventive Veterinary Medicine in Hubei Province The Cooperative Innovation Center for Sustainable Pig Production, Wuhan, Hubei, China; ^4^ College of Henan Science and Technology, Xinxiang, Henan, China; ^5^ State Key Laboratory of Veterinary Etiological Biology National Foot-and-Mouth Disease Reference Laboratory Lanzhou Veterinary Research Institute Chinese Academy of Agricultural Sciences, Lanzhou, Gansu, China

## Abstract

Seneca Valley virus (SVV) and foot-and-mouth disease virus (FMDV) belong to the Picornaviridae family, which can cause similar symptoms. After infection, pigs will develop fever; loss of appetite; blister lesions on the skin and mucous membrane of the mouth, nose, and hoof; and other similar diseases, and the spread is very fast, causing major economic losses to the pig industry. Therefore, a rapid, accurate, and sensitive diagnostic method is necessary to enable rapid prevention and control measures for preventing the spread of these diseases. Here, a fluorescent immunochromatography test strip, using Eu-doped fluorescence beads and monoclonal antibody, was developed for the simultaneous determination of FMDV and SVV. The test process for the assay could be completed in 12 min, which avoided the time cost of the current methods for FMDV/SVV detection. Under optimized conditions, the limit of detection of SVV is 5 × 10^4^ PFU/mL, and that of FMDV is 5 × 10^4^ PFU/mL under the Fluorescence Immunoassay Analyzer. Our assay results showed a good linear correlation with RT-PCR installed in the clinical laboratory. The species design has a promising application prospect in the surveillance and control of the outbreak of idiopathic blister.

## 1. Introduction

Seneca Valley virus (SVV) and foot-and-mouth disease virus (FMDV) are important pathogens of idiopathic vesicular disease, both belonging to the Picornaviridae family. Animals infected SVV or FMDV have very similar symptoms, such as fever, anorexia, and blisters or ulcerations on the nose, mouth, and hooves [1, 2], which seriously affects the healthy development of animal husbandry.

SVV is the only member of the genus *Senecavirus* of the family Picornaviridae with a genome size of ~7.2 kb [3]. After SVV infection, obvious blister-like lesions could be seen in the mouth, nose, and hoof of pigs. The disease is extremely harmful to newborn piglets. Studies have reported that SVV infection can lead to acute death of newborn piglets, and its fatality rate can reach 30%–70% [4]. Since SVV was first identified in PER.C6 cell line culture contaminants in 2002 [5], it has spread rapidly throughout the world. In 2014, a large outbreak of SVV occurred in Brazil and then took place in the United States, Canada, Colombia, Thailand, China, and Vietnam [6]. In 2015, SVV was detected in a pig farm in Guangdong Province, China. Subsequently, SVV infection was reported in many provinces, including Heilongjiang, Henan, Hubei, and so on [7]. It is a serious threat to the development of the global pig industry.

FMDV belongs to the genus of foot-and-mouth disease virus in the family Picornaviridae. Its infection mainly causes acute and virulent infectious diseases mainly against even-toed ungulates [8]. After FMDV infection, animals develop symptoms such as high fever, severe emaciation, and blister lesions in the mouth and hooves. In addition, FMDV can also cause an increase in mortality of young animals, a decrease in maternal fertility, and barriers to international trade in animals and animal products. FMDV can pollute the environment through aerosols, leading to long-distance transmission events, which seriously threatens the development of animal husbandry and international trade. Therefore, it is also classified as a group A severe animal infectious disease by the World Organization for Animal Health (WOAH). There are seven serotypes of FMDV, but there is no effective cross-protection between different serotypes, and each serotype can undergo a large degree of mutation [9], which makes the prevention and control of foot-and-mouth disease more difficult.

Animals infected with SVV and FMDV will have similar vesicular lesions, and the symptoms are very similar. It is difficult to distinguish between SVV and FMDV by clinical diagnosis alone. At present, the two viruses are mainly diagnosed by virus isolation, RT-PCR, qRT-PCR, ELISA, and indirect immunofluorescence [6, 7, 10, 11]. These methods are inadaptable for point-of-care detection because they require long detection time, expensive instruments, and professional operators. Therefore, a rapid, simple, and accurate detection method to identify the two pathogens is of great significance for the control and prevention of idiopathic vesicular disease.

Immunochromatography test strip (ITS) has been widely used in point-of-care detection due to their outstanding properties such as rapid process, no washing and incubation steps, and lack of skilled technicians and precision instrument [12, 13, 14]. However, traditional colloidal Au-based immunochromatography test strip is limited applicated in early detection due to low detection sensitivity [15]. Great efforts have been made toward developing nanomaterials, and several alternative label nanomaterials have been introduced, such as magnetic beads, upconverting phosphors, colored latex nanoparticle, quantum dots (QDs), and time-resolved fluorescence beads [16, 17, 18]. Eu-doped fluorescence beads as fluorescent labels have attracted great interest in medical diagnose because of good optical properties such as long fluorescence lifetimes and narrow photoluminescence. Besides, good colloidal stability makes them suitable for surface functionalization [13].

In this study, an Eu-doped fluorescence bead-based immunochromatography test strip (FITS) was established for simultaneous detection of FMDV and SVV. The test results can be qualitatively analyzed by the naked eye under the irradiation of the portable ultraviolet (UV) lamp. Besides, the FITS has shown high sensitivity, the limit of detection of SVV is 5 × 10^4^ PFU/mL, and that of FMDV is 5 × 10^4^ PFU/mL under the Fluorescence Immunoassay Analyzer, as it is similar to the conventional RT-PCR. Our FITS has promising application prospects in the surveillance and control of idiopathic blister disease outbreaks.

## 2. Materials and Methods

### 2.1. Materials and Reagents

SVV monoclonal antibodies (mAb) 6D7 and 5G6 were prepared in our laboratory. Japanese encephalitis virus (JEV), porcine reproductive and respiratory syndrome virus (PRRSV), porcine pseudorabies virus (PRV), porcine circovirus type 2 (PCV2), and SVV were prepared and stored in our laboratory. FMDV mAb 1C6 were presented by the Lanzhou Veterinary Research Institute, Chinese Academy of Agricultural Sciences. FMDV was maintained in the National Foot-and-Mouth Disease Reference Laboratory, Lanzhou Veterinary Research Institute, Chinese Academy of Agricultural Sciences. Inactivated FMDV O/HN/CHA/93 cell culture supernatants were used as FMDV-positive samples in the experiment. Viral titers were measured prior to inactivation. These inactivated FMDV-positive samples were provided by the National Foot-and-Mouth Disease Reference Laboratory, Lanzhou, China.

Nitrocellulose membrane (Vivid 120), sample pad, conjugation pad (GL-b04), absorptive pad (H5072), and plastic casings were purchased from Shanghai Jenin Biotechnology (Shanghai, China). Eu-doped fluorescence beads (FB, 300 nm, 10 mg/mL) and Alexa Fluor 488 goat antimouse antibody were purchased from Thermo Fisher Scientific (USA). Bovine serum albumin (BSA), N-hydroxysuccinimide (NHS), 1-ethyl-3- (3-dimethyl aminopropyl) carbodiimide hydrochloride (EDC), morpholine ethyl sulfonic acid (MES), adjuvant (Freund's complete and incomplete), PEG 4000, HAT, and HT were purchased from Sigma–Aldrich (USA). Rabbit antimouse IgG were purchased from Proteintech (USA). Goat antipig IgG were purchased from ImmunoWay (USA). HRP rapid labeling kit was purchased from Bioablab (Luoyang, China). DL2000TM DNA Marker was purchased from TaKaRa (Daliang, China). The sequences (5′−3′) of specific PCR primers were as follows: SVV-F, ATTCCTGCGTCGCCAAAG, and SVV-R, ACGAATCGTAAACACCATTGTTCAC; and FMDV-F, GAGGCCAAACCCTGGTACAAG; and FMDV-R, TTGATGTCACGTGCTTTGAG. They were synthesized from Sangon Biotech (Wuhan, China).

### 2.2. Instrumentation

We used a H-7650 transmission electron microscope (Japan, HITACHI), JSM-6390LV scanning electron microscopy (Japan, NTC), and Nano ZS Laser Particle Size and Zeta Potential Analyzer (UK, Malvern) to characterize and analyze the prepared fluorescent probes. CM4000 Guillotine Cutter (USA, Biodot) and XYZ3050 dispensing platforms (USA, Biodot) were used to prepare FITS. MX-RD-E Rotary Mixer, MX-S Vortex Mixer (China, Dragon), and 5,425 R centrifuge (Germany, Eppendorf) were used to prepare fluorescence bead-labeled monoclonal antibody (FB-mAb). The fluorescence intensity on the fluorescence immunochromatography test strip was read by AFS-1000 Fluorescence Immunoassay Analyzer (China, LABSIM). A PCR amplification apparatus (Germany, Eppendorf) was used to amplify cDNA fragments. ChemiDoc XRS gel imaging system (USA, BIO-RAD) and DYCP-31CN Agarose Horizontal Electrophoresis Apparatus (China, Liuyi) were used to analyze the PCR products.

### 2.3. Preparation of the Corresponding Antibodies

We infected BHK-21 cells with SVV HB-CH-2016 and repeatedly freeze-thawed the cells three times when they became diseased. Cell debris was then removed by centrifugation at 4°C and 6,010 rcf (g) for 30 min. We concentrated the virus by ultracentrifugation; the bottom of the tube was cushioned with 20% sucrose during centrifugation. We then resuspended the concentrated virus in PBS after ultracentrifugation. After concentration, the virus was further purified by 20%–65% sucrose gradient centrifugation. Then, the virus band was taken for desucrose treatment, and the precipitate was resuspended in PBS and stored at −80°C. The purified SVV HB-CH-2016 was used as antigen to immunize mice. Forty microgram of SVV HB-CH-2016 was emulsified with an equal volume of Freund's complete adjuvant and injected subcutaneously into 6-week-old female Balb/c mice. After the first immunization, mice were immunized twice with the same dose of SVV HB-CH-2016 and the same volume of Freund's incomplete adjuvant at a 2-week interval. After the third immunization of the mice, the immune efficacy was determined by ELISA, and the mice with high immune titer were given a booster immunization. Three days after booster immunization, the Balb/c mouse was killed by cervical dislocation, and then mouse spleen cells were collected and fused with 50% PEG4000 to SP2/0 cells. Screening passaging culture in fused cells with HAT-containing medium. The hybridoma cell line secreting monoclonal antibody was screened by ELISA and subcloning by limiting dilution method.

### 2.4. Purification of Antibodies and Their Detection Efficiency

The antibody titer was tested by indirect ELISA assay.

Monoclonal antibodies were prepared by inducing ascites in mice. After waiting for mouse belly swelling dramatically, the mice were killed by cervical dislocation, and then ascites were collected. Using protein A/G column purification of antibodies from the ascites, the purified SDS-PAGE of ascites were identified. Identify antibody specificity using indirect immunofluorescence. BHK-21 cells were cultured in 24-well plates; when the cells are full of a single layer, the cells were inoculated with 0.1 MOI SVV HB-CH-2016. After 12 hr, the cells were fixed with methanol for 20 min at −20°C, washed three times with PBS, and blocked for 1 hr with PBS containing 2% BSA and 0.3% Triton X-100. The prepared purified antibody was then added to the culture wells, incubated at 37°C for 1 hr, then washed three times with PBS, added with Alexa Fluor 488 goat antimouse antibody, and incubated at room temperature for 1 hr. The results were observed under a fluorescence microscope.

HRP labeling of antibodies with high ELISA potency was done using the HRP Rapid Labeling Kit. The mAb pairs for SVV detection were selected by sandwich ELISA.

### 2.5. Fluorescence Bead Surface Functionalization

First, 10 *μ*L of fluorescence beads and 990 *μ*L of MES were added, and then let them mixed well. Then, 10 *µ*L each of EDC (10 mg/mL) and NHS (50 mg/mL) was prepared with MES and was added and stirred in the dark for 30 min at room temperature to activate the carboxyl groups on the surface of the fluorescence beads. The activated fluorescence beads were centrifuged at 21,130 rcf (g) for 30 min, the supernatant was discarded, and the activated fluorescence beads were resuspended by adding 1 mL of MES. Then, add 5 *μ*L of 5G6 (1 mg/mL), and incubate for 3 hr; the reaction was blocked by 10 *μ*L of 10%BSA for 1 hr. Centrifuging at 4°C, 21,130 rcf (g) for 15 min, washing with PBS three times and resuspending with 1 mL of storage solution after the blocking reaction, the product was stored in dark at 4°C for later use.

The 1C6 fluorescence bead conjugate was prepared as described above. A 7 *μ*L of 1C6 (1 mg/mL) was added to the reaction for 3 hr and then blocked by the same method mentioned prior. The final product was resuspended in 1 mL of storage solution and stored at 4°C in the dark until use.

### 2.6. FITS Preparation

The FITS consisted of four parts: sample pad, conjugate pad, NC membrane, and absorptive pad. A dispenser system was used to load a set volume of the antibody fluorescence bead conjugate to the conjugation pad. And the NC membrane was prepared by dispensing 0.6 mg/mL 6D7 or 0.6 mg/mL 1C6 to the test lines (T-line) onto the NC membrane. The 0.3 mg/mL rabbit antimouse IgG were dispensed on specific areas of the NC membrane called the control line (C-line). Since 1C6 is of porcine origin, the antibody on the C-line of FMDV-FITS is goat antipig IgG. The treated conjugation pad and the NC membranes were dried at 37°C. Then, the FITS were cut into individual 4-mm-wide strips mounted in plastic case and stored in a sealed bag with desiccant for later use.

### 2.7. Sensitivity and Specificity of FITS

To test the sensitivity of FITS, SVV was diluted in PBS to a series of concentrations and prepared as positive reference samples. Then, 75 *μ*L of each sample was added into the sampling wells of SVV single-component FITS in turn, and the fluorescence intensity was detected after 12 min of reaction, and the standard curve was drawn. Similarly, FMDV was diluted with PBS into a series of concentrations of positive reference samples, which were then detected with FMDV single-component FITS, and a standard curve was drawn. At the same time, RT-PCR was used to detect different dilutions of virus compared with FITS.

SVV and FMDV positive reference was mixed, and 75 *μ*L of it as a sample was dropped into the sampling wells of SVV-FMDV multiplex FITS. After 12 min, the fluorescence intensity on the T1 and T2 lines was measured.

The specificity of FITS was evaluated, and the specificity of FITS was tested by PRRSV, PCV2, JEV, and PRV. The above viruses were diluted to the same concentration (10^6^ PFU/mL) with PBS, and 75 *μ*L of each was as a sample dropping into FITS. After 12 min, the fluorescence intensity was detected by Fluorescence Immunoassay Analyzer.

### 2.8. Detection of Samples

We collected a total of 80 samples. In SVV-infected pigs, we used cotton swabs to dip blister fluid or collected vesicular epithelium and soaked it in PBS to thoroughly mix, and 32 of the mixture were used for detection. The remaining 48 samples were cell supernatants collected after inoculation of SVV-positive disease material into cells. We tested these 80 samples using FITS and routine laboratory RT-PCR.

### 2.9. Statistics

The error bar represents the standard deviation (*n* = 3). All data were analyzed for statistical significance using the Student's *t* test. *P* < 0.05 was considered statistically significant, and the *P* value was represented as follows:  ^*∗*^*P* < 0.05;  ^*∗∗*^*P* < 0.001; and  ^*∗∗∗*^*P* < 0.0001.

## 3. Results

### 3.1. Preparation, Purification, and Indirect Immunofluorescence Analysis of the Corresponding Antibodies

After cell fusion, hybridoma cell lines 5G6, 6D7, and 3F11 with high ELISA efficacy were screened, and mice were immunized to produce ascites (Figure S1). Purification of antibodies from ascites using protein A/G columns yielded three strains of antibodies 3F11, 6D7, and 5G6 with high purity (Figure S2). After IFA assay, all three purified antibody strains were able to bind specifically to SVV (Figure S3). The optimal detection of the two antibody pairs, 5G6 and 6D7, was verified by both sandwich ELISA and fluorescence immunochromatographic methods (Figures S4 and S5).

MAb 1C6 was prepared based on single B-cell antibody technology and was gifted by Lanzhou Veterinary Research Institute, Chinese Academy of Agricultural Sciences. 1C6 cross-reacted with FMDV serotype A and O strains. Li et al. [19] detected that 1C6 was able to specifically recognize VP2 of type A and O FMDV by Western blotting, indicating that the mAb 1C6 has broad-spectrum activity against FMDV serotype A and O strains. In 1958, FMD were first reported in China. Then three serotypes of FMDV, including O, A, and Asia-1, have been endemic in China. However, FMDV of serotype Asia-1 has not been reported in China in the past 15 years [20]. Therefore, 1C6 is able to meet the testing requirements for FMD in China.

### 3.2. The Mechanism of FITS

**Figure 1:**
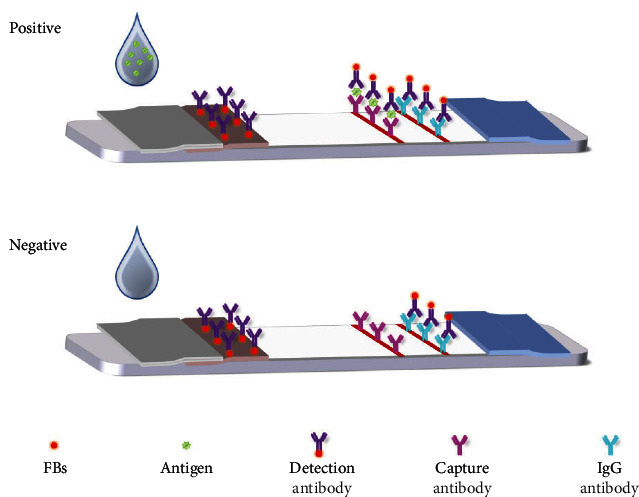
Schematic diagram of fluorescence immunochromatographic test strips.

The mechanism of FITS is based on the reaction of antibody with antigen, which forms a sandwich structure with the antigen (Figure 1). After successful modification of the detection antibody on the surface of the fluorescence beads, they were immobilized as fluorescent probes on a conjugate pad. And then the capture antibody and rabbit antimouse IgG (or goat antipig IgG) were coated on the T-line and C-line of the NC membrane, respectively. When the sample contains the target antigen (SVV/FMDV), the target antigen specifically binds to the detection antibody on the conjugated pad, forming an antigen–antibody immune complex. The immune complex under the action of capillary flows forward, and when it flowed to the T-line, with the capture antibody sandwich structure formation, it produces positive results. The Immunochromatography test strip was able to see red fluorescent bands on the T- and C-lines under UV light. When there are no SVV/FMDV in samples, unable to form immune complex, and not with the capture antibody, T-line cannot see the fluorescence; it presents the negative result. In both cases, the excess fluorescent probe conjugate would continue to flow forward under capillary action to bind rabbit antimouse IgG (or goat antipig IgG) on the C-line, and thus, fluorescence would be seen at the C-line position. Under the UV lamp, we can qualitatively analyze the samples with the naked eye. The antigen in the sample can be quantified by reading the fluorescence intensity of FITS using Fluorescence Immunoassay Analyzer.

### 3.3. Functionalization of Fluorescence Beads

**Figure 2:**
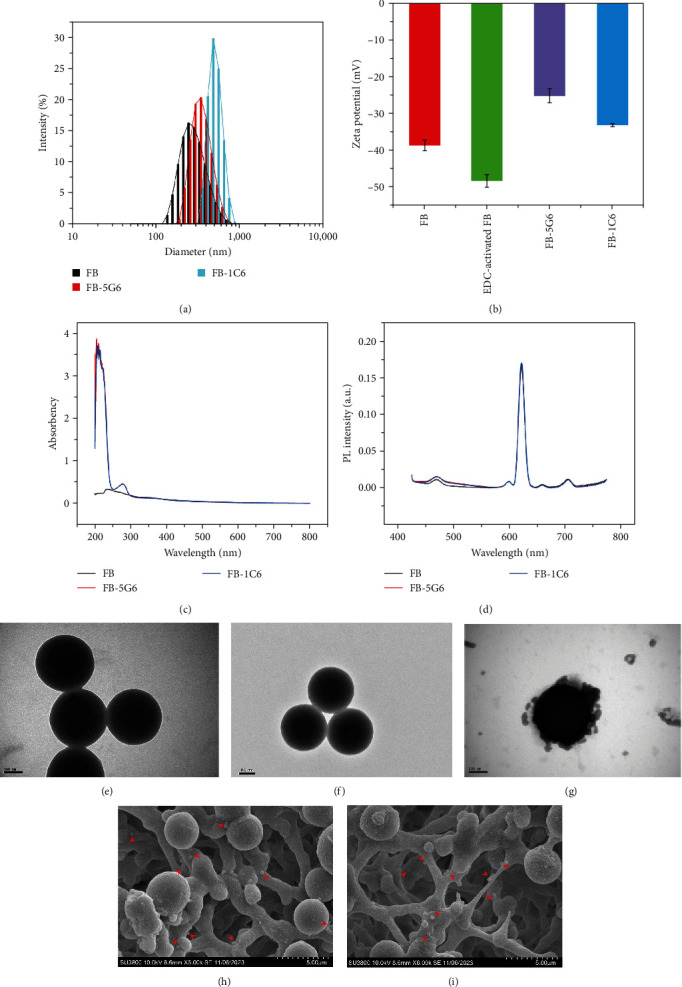
Functionalization of fluorescence beads: (a) changes in particle size of fluorescence beads before and after antibody labeling; (b) surface zeta potential of fluorescence beads (FB), activated fluorescence beads (EDC-activated FB), fluorescence bead 5G6 conjugate (FB-5G6), and fluorescence bead 1C6 conjugate (FB-1C6); (c) UV–vis absorption spectra of FB, FB-5G6, and FB-1C6; (d) PL emission spectra of FB, FB-5G6, and FB-1C6; (e) TEM images of fluorescence beads. Scale bar = 100 nm. (f) TEM images of FB-5G6. Scale bar = 100 nm. (g) TEM images of SVV bound to the FB-5G6. Scale bar = 100 nm. (h) SEM image of the FITS T-line in the presence of SVV. Scale bar = 5.00 *μ*m. (i) SEM image of the FITS T-line in the presence of FMDV. Scale bar = 5.00 *μ*m.

We performed dynamic scattering measurements of the hydrodynamic diameter and indicative charge of the fluorescence beads after antibody modification using a Malvern nanolaser granulometer. Results show that the particle size of fluorescence beads modified by antibody increased slightly (Figure 2(a)). Zeta potential analysis results show that the negative charge on the surface of the fluorescence beads after the EDC activating increased slightly. And beads after antibody labeling, because of the antibody coupling with the carboxyl group on the surface of the beads, the negative charge of the beads corresponding decreases (Figure 2(b)). We also explored the UV–vis absorption and fluorescence emission spectra of fluorescence beads before and after labeling antibodies. As shown in Figures 2(c) and 2(d), UV–vis absorption spectra of fluorescence beads after labeling antibodies 5G6 and 6D7 showed a clear antibody absorption peak near 280 nm. The fluorescence bead emission peak was centered at 625 nm, and there was no significant change after labeling the antibody. TEM results showed that the fluorescence beads after labeling antibody diameter were slightly larger than that of the fluorescence beads of the unmodified antibody, and the fluorescence beads after labeling with the 5G6 antibody were able to bind to SVV (Figures 2(e), 2(f), and 2(g)). After, we observed that the FB-5G6 were surrounded by SVV virions with a diameter of ~30 nm (Figure 2(g)). In conclusion, it can be judged that the antibody was successfully coupled to the surface of the fluorescence beads.

To determine whether FB-5G6 or FB-1C6 was captured on the T-line, we measured SEM images of T-line. As shown in Figure 2(h), we observed immune complexes in the T-line region, which were formed by SVV binding to FB-5G6 and being captured by 6D7 (the round particles indicated by red arrows are “FB-5G6-SVV-6D7” immunocomplexes). In the presence of FMDV, we also observed immune complexes in the T-line region of the NC membrane (the “FB-1C6-FMDV-1C6” immune complexes are indicated by the red arrow in Figure 2(i)). These results indicate that FITS is capable of diagnosing the presence of SVV and FMDV.

### 3.4. Optimization of FITS

To achieve optimal FITS performance, the amount of antibody labeling, antibody concentration on the T-line, and the concentration of fluorescent probe and reaction time were optimized. The result showed that 5 *μ*g of 5G6 and 7 *μ*g of 1C6 were the optimal amount of antibody labeling (Figure S6). The 0.6 mg/mL was the best concentration on the T-line antibody (Figure S7). The optimal concentrations of fluorescent probes FB-5G6 and FB-1C6 were both 5 *μ*g/mL (Figure S8). And 12 min of reaction time was the optimal condition (Figure S9).

### 3.5. Sensitivity and Specificity of FITS

**Figure 3:**
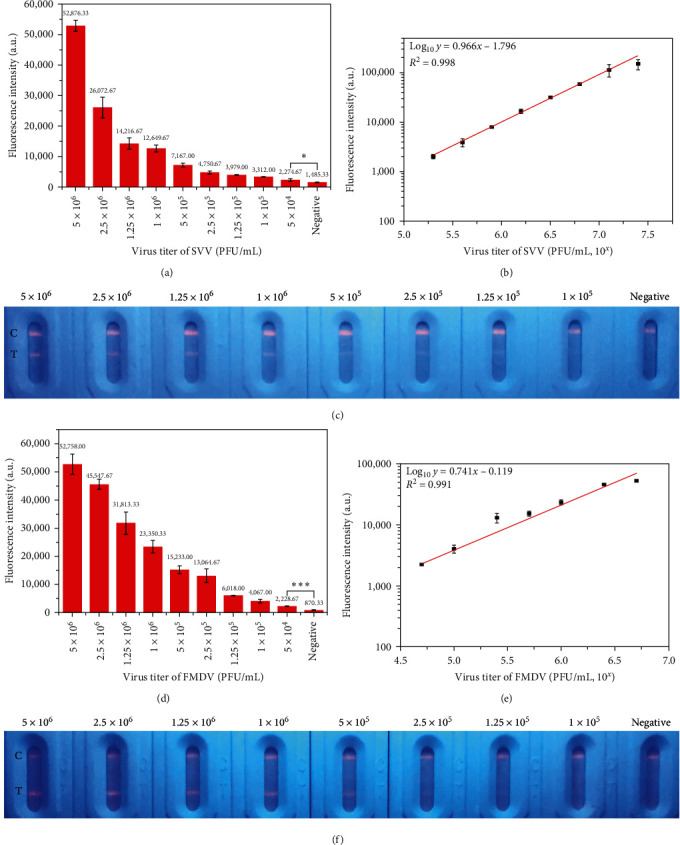
Sensitivity of FITS: (a) fluorescence intensity of the T-line at different virus titers of SVV by Fluorescence Immunoassay Analyzer. Each value represents the mean of three replicates (*n* = 3). The data between the 5 × 10^4^ PFU/mL of SVV positive samples and negative samples were compared with the Student's *t* test ( ^*∗*^*P* < 0.05). (b) The standard curve of SVV-FITS. Each value represents the mean of three replicates (*n* = 3). (c) Image of the SVV-FITS at different virus titers of SVV by UV lamp. (d) Fluorescence intensity of the T-line at different virus titers of FMDV by Fluorescence Immunoassay Analyzer. Each value represents the mean of three replicates (*n* = 3). The data between the 5 × 10^4^ PFU/mL of FMDV positive samples and negative samples were compared with the Student's *t* test ( ^*∗∗∗*^*P* < 0.0001). (e) The standard curve of FMDV-FITS. Each value represents the mean of three replicates (*n* = 3). (f) Image of the FMDV-FITS at different virus titers of FMDV by UV lamp.

Under optimized conditions, we tested the sensitivity and specificity of FITS. The positive samples of SVV and FMDV were diluted into different concentrations for quantitative detection. The quantitative detection was to evaluate the ability and sensitivity of FITS. At the same time, different concentrations of virus were detected by conventional RT-PCR in our laboratory and compared with FITS. As shown in Figures 3(a) and 3(c), the fluorescence intensity on the T-line decreased with the decrease of SVV virus titer, and the T-line gradually became fainter and fainter as seen by the naked eye under ultraviolet light. The visible qualitative detection limit of SVV-FITS is 1 × 10^5^ PFU/mL. And the lowest detection limit of SVV-FITS under the Fluorescence Immunoassay Analyzer is 5 × 10^4^ PFU/mL. We also found that when the virus titer of SVV was in the range of 10^5.3^ to 10^7.4^ PFU/mL, there was a good linear relationship between the fluorescence intensity on the T-line and the virus titer (Figure 3(b)). We fitted a linear equation with virus titer as the horizontal coordinate and T-line fluorescence intensity as the ordinate. The equation was Log_10_*y* = 0.966*x* − 1.796, and *R*^2^ = 0.998. Conventional RT-PCR results showed (Figure S10) the specific bands of SVV were close to disappearing when the viral titer of SVV was 10^5^ PFU/mL. It indicated that the limit of SVV-FITS reached that of conventional RT-PCR.

And as shown in Figures 3(d) and 3(f), the fluorescence intensity gradually decreased, and T-line became blurred and indistinct with the decrease of FMDV virus titer. When the virus titer of FMDV is 1 × 10^5^ PFU/mL, the changes in the T-line were difficult to observe by the naked eye. And the lowest detection limit of FMDV-FITS under the Fluorescence Immunoassay Analyzer are 5 × 10^4^ PFU/mL. The detection limit of FMDV-FITS was similar to conventional RT-PCR (Figure S11). The linear detection range of FMDV-FITS was 10^4.7^−10^6.7^ PFU/mL and the equation of FMDV-FITS was Log_10_*y* = 0.741*x* − 0.119, and *R*^2^ = 0.991 (Figure 3(e)).

**Figure 4:**
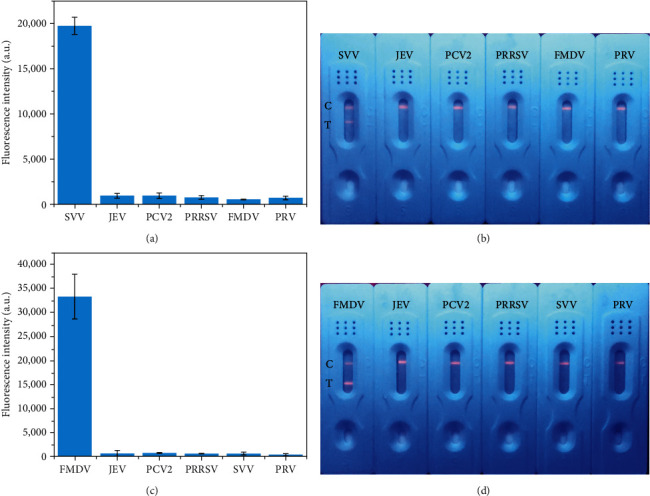
Specificity of FITS: (a) Fluorescence intensity on the SVV-FITS T-line for the detection of different viruses. Each value represents the mean of three replicates (*n* = 3). (b) Image of the SVV-FITS at detecting different viruses by UV lamp. (c) Fluorescence intensity on the FMDV-FITS T-line for the detection of different viruses. Each value represents the mean of three replicates (*n* = 3). (d) Image of the FMDV-FITS at detecting different virus by UV lamp.

At the same time, we also tested the JEV, PCV2, PRRSV, and PRV, to evaluate the SVV-FITS and FMDV-FITS specificity. The results are shown in Figures 4(a) and 4(b), in SVV-FITS, except for the fluorescence signal of detected SVV, which reached a high level, the fluorescence signal of other virus samples was close to the blank value. In FMDV-FITS (Figures 4(c) and 4(d)), only FMDV could be detected, and the rest of the virus samples were negative. These results indicate that both SVV-FITS and FMDV-FITS prepared have good specificity.

### 3.6. Multiplexing Detection of SVV and FDMV by FITS

**Figure 5:**
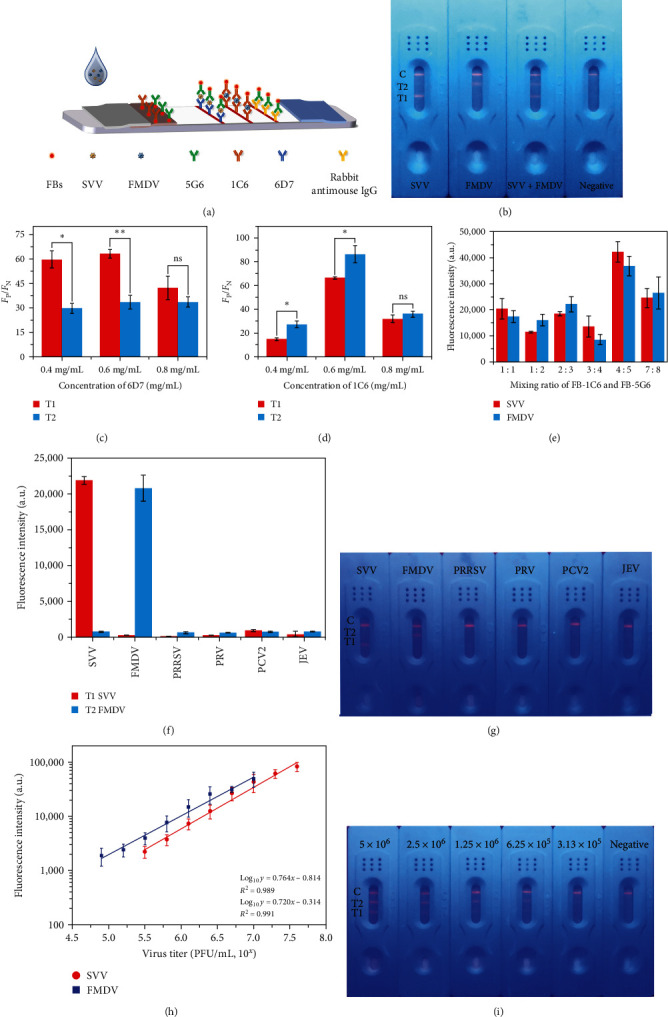
Multiplexing detection of SVV and FDMV by FITS: (a) schematic of multiplexed FITS fabrication for simultaneous detection of FMDV and SVV, (b) images of the resulting SVV and FMDV detected by multiplex FITS, and (c) the effect of 6D7 concentration and position on the fluorescence intensity of the T-line was examined using SVV-positive and SVV-negative samples. Each value represents mean value of *F*_P_/*F*_*N*_ obtained from three assays. The data between the T1 and T2 were compared with the Student's *t* test ( ^*∗*^*P* < 0.05,  ^*∗∗*^*P* < 0.001). (d) The effect of 1C6 concentration and position on the fluorescence intensity of the T-line was examined using FMDV-positive and FMDV-negative samples. Each value represents mean value of *F*_P_/*F*_*N*_ obtained from three assays. The data between the T1 and T2 were compared with the Student's *t* test ( ^*∗*^*P* < 0.05). (e) Effect of mixing ratio on the detection ability of multiplex FITS. (f) Fluorescence intensity on the multiplex FITS T-lines for the detection of different viruses. Each value represents the mean of three replicates (*n* = 3). (g) Images of multiplex FITS at detecting different viruses by UV lamp. (h) Standard curves for multiplex FITS. (i) Image of the multiplex FITS at different virus titers of SVV and FMDV by UV lamp.

Animals infected with SVV or FMDV develop similar blistering lesions and have very similar symptoms, which are difficult to distinguish by clinical diagnosis alone. In order to realize the simultaneous detection of SVV and FMDV-based FITS, the corresponding capture antibodies of these two viruses were immobilized on the same test strip; fluorescent probes for SVV and FMDV (FB-5G6 and FB-1C6) were mixed and immobilized on conjugate pads, as shown in Figure 5(a). For multiple immunochromatography, the binding line that locates different specific reactants is the best solution to solve the change of reactant concentration in solution with the flow of the solution in the test strip in multiple immunochromatography [21].

We optimized the concentration and line position of the two capture antibodies in the multiplex fluorescence immunochromatography system. The results showed that when the concentration of 6D7 was 0.6 mg/mL at the T1-line position, and the concentration of 1C6 was 0.6 mg/mL at the T2-line position, the difference between the negative samples and the positive samples was the largest (Figures 5(c) and 5(d)). Then we optimized the mixing ratio of the two detection antibody bead conjugates, and the results showed that when the mixing ratio of FB-1C6 and FB-5G6 was 4 : 5, the detection effect of the two T-lines was the best (Figure 5(e)). We also evaluated the cross-reactivity of the multiplex immunochromatographic system. As shown in Figure 5(b), we clearly observed that when SVV was present in the sample, only T1-line showed strong positive fluorescence signal, but T2-line was negative. When FMDV was present in the sample, T2-line was positive, but the T1-line fluorescence signal was close to the blank value. This shows that both above antibodies have specificity and are low cross-interference. We also mixed SVV and FMDV for detection to further test the feasibility of the multiple immunochromatographic system. The result shown in Figure 5(b) is that both the detection lines showed fluorescence signals.

We performed specific detection of the multiplex FITS using different viruses, and the results are shown in Figures 5(f) and 5(g). Except for the strong fluorescent signals on the corresponding T-lines when SVV and FMDV were detected, no fluorescence appeared on T1-line and T2-line when other viruses were detected.

Then, we also performed a gradient dilution of the virus to examine the effect of the multiplex immunochromatographic system for SVV and FMDV detection at different virus titers (Figure 5(i)). We found that when the virus titer of SVV was in the range of 10^5.5^−10^7.6^ PFU/mL and the virus titer of FMDV was in the range of 10^4.9^−10^7^ PFU/mL, there was a linear relationship between the virus titer and the fluorescence intensity on the corresponding T-lines when detected by multiplex FITS. For this purpose, we established standard curves (Figure 5(h)). The equation of the standard curve of SVV was Log_10_*y* = 0.764*x* − 0.814 (*R*^2^ = 0.989). The equation of the standard curve of FMDV was Log_10_*y* = 0.720*x* − 0.314 (*R*^2^ = 0.991). These results indicate that the multiplexing detection of SVV and FDMV by FITS is feasible. This assay has a great prospect in the rapid differentiation of SVV and FMDV in clinical practice in the future.

### 3.7. Detection of Samples

We used FITS to detect samples from 80 clinical samples with and compared the results with RT-PCR. As shown in Table 1, FITS compared with RT-PCR, the positive coincidence rate of SVV detection was 97.1%, the negative coincidence rate was 100%, and the total coincidence rate was 97.5%.

## 4. Discussion

In this study, we established a FITS for the rapid and visual detection of SVV and FMDV. Under the optimal detection conditions, only 75 *μ*L blister fluid sample was needed to complete the detection within 12 min. Our established FITS showed high specificity for both SVV and FMDV. Compared to RT-PCR, it offers good consistency and greatly reduced assay time and does not require specialized equipment or complicated handling. More importantly, we successfully realized multiplexed detection of multiple blister disease pathogens by using this FITS. This method is appropriate for point-of-care testing in low-resource areas and has potential for surveillance, prevention, and control of vesicular diseases. We expect that this method can be widely used in the clinic for early diagnosis or regular monitoring of vesicular diseases in the future.

Animals infected with SVV and FMDV will have similar vesicular lesions, and the symptoms are very similar. It is difficult to distinguish between SVV and FMDV by clinical diagnosis alone. In this study, we established a FITS based on Eu-doped fluorescence beads for rapid and visual distinguishes detection of SVV or FMDV. Compared with other test method such as virus isolation, RT-PCR, qRT-PCR, ELISA, and indirect immunofluorescence, this method is more rapid and simple. Under the optimal detection conditions, only 75-*μ*L blister fluid sample was needed to complete the detection within 12 min. In addition, the FITS has shown high specificity and sensitivity. There no cross-reactivity with PRRSV, PRV, PCV2, and JEV. Besides, the limit of detection of SVV is 5 × 10^4^ PFU/mL, and that of FMDV is 5 × 10^4^ PFU/mL, as it is similar to the conventional RT-PCR. However, there are still some limitations to this FITS. The FITS are not suitable for mass screening.

In summary, a novel FITS based on Eu-doped fluorescence beads has been successfully established for the rapid and visual detection of SVV and FMDV. The assay not only specificity recognizes the SVV or FMDV but also has the ability to differentially diagnose SVV and FMDV. The assay was highly specific for both SVV and FMDV, and the results of the FITS were in good agreement with those of RT-PCR. Therefore, the assay is appropriate for point-of-care detection in low-resource areas and has potential for surveillance, prevention, and control of vesicular disease. We believe that the newly developed assay has potential in the clinic for early diagnosis or regular monitoring of vesicular diseases.

## Figures and Tables

**Table 1:** The total coincidence rate of RT-PCR and the FITS detection.

Analytical methods	FITS		Total
	Positive	Negative	
RT-PCR			
Positive	67	0	67
Negative	2	11	13
Total	69	11	80
Coincidence rate (%)	97.1%	100%	97.5%

## Data Availability

Data sharing not applicable to this article as no data sets were generated or analyzed during the current study.

## Supplementary Materials

Supplementary Materials Figure S1: determination of ELISA titer of hybridoma cell supernatant. Figure S2: the purified antibody was detected by SDS-PAGE. Figure S3: identify antibody specificity using indirect immunofluorescence. Figure S4: detection of antibody pairs by sandwich ELISA. Figure S5: detection of antibody pairs by sandwich FITS. Figure S6: optimization of the amount of antibody labeling. Figure S7: optimization of antibody concentration on the T-line of FITS. Figure S8: optimization of fluorescent probe concentration. Figure S9: optimization of reaction time of FITS. Figure S10: results of conventional RT-PCR for SVV. Figure S11: results of conventional RT-PCR for FMDV.
