# Supplementary material for: A Fluorescent Immunochromatography Test Strip for the Rapid Identification of SVV and FMDV
Source: Transbound Emerg Dis. 2024 Aug 12;2024:1628008. doi: 10.1155/2024/1628008 (PMC12016916; doi:10.1155/2024/1628008)
Supplement: Supplementary Materials — Figure S1: determination of ELISA titer of hybridoma cell supernatant. Figure S2: the purified antibody was detected by SDS-PAGE. Figure S3: identify antibody specificity using indirect immunofluorescence. Figure S4: detection of antibody pairs by sandwich ELISA. Figure S5: detection of antibody pairs by sandwich FITS. Figure S6: optimization of the amount of antibody labeling. Figure S7: optimization of antibody concentration on the T-line of FITS. Figure S8: optimization of fluorescent probe concentration. Figure S9: optimization of reaction time of FITS. Figure S10: results of conventional RT-PCR for SVV. Figure S11: results of conventional RT-PCR for FMDV. [file 1628008.f1.docx]

**Supplementary Information**

**A fluorescent immunochromatography test strip for the rapid identification of SVV and FMDV**

Liuyue Yang^a,b,c#^, Chengfei Li^a,b,c,e#^, Xinghua Chen^a,b,c^, Kun Li^d^, Zengjun Lu ^d^, Xiangmin Li^a,b,c^, Meilin Jin^a,b,c*^ and Ping Qian^a,b,c *^

^a^National Key Laboratory of Agricultural Microbiology, Hubei Hongshan Laboratory, Huazhong Agricultural University, Wuhan 430070, Hubei, China

^b^Laboratory of Animal Virology, College of Veterinary Medicine, Huazhong Agricultural University, Wuhan 430070, Hubei, China

^c^Key Laboratory of Preventive Veterinary Medicine in Hubei Province, The Cooperative Innovation Center for Sustainable Pig Production, Wuhan, Hubei, China

^d^State Key Laboratory of Veterinary Etiological Biology, National Foot-and-Mouth Disease Reference Laboratory, Lanzhou Veterinary Research Institute, Chinese Academy of Agricultural Sciences, Lanzhou, Gansu, People’s Republic of China

^e^College of Henan Science and Technology, Xinxiang, Henan, China.

^#^These authors contributed equally to this work

*Correspondence: [qianp@mail.hzau.edu.cn](mailto:qianp@mail.hzau.edu.cn); [jinmeilin@mail.hzau.edu.cn](mailto:jinmeilin@mail.hzau.edu.cn)

**Materials and methods**

**1 Optimization of FITS**

**1.1 Optimization of antibody labeling amount**

We labeled fluorescent beads with SVV monoclonal antibody 5G6 of different amounts (2, 4, 5, 6 and 8 μg) to prepare the fluorescent probe FB-G6. Then we prepared SVV-FITS according to the FITS preparation method. The SVV virus solution was then diluted with PBS to 10^7^ PFU/mL as a positive sample, while PBS was used as a negative control. 75 μL of negative sample and positive sample were added to SVV-FITSs and after reaction for 12 min the fluorescence intensity was detected by Fluorescence Immunoassay Analyze. Each test was performed three times, the fluorescence intensity of the positive and negative samples was recorded and the ratio of the fluorescence intensity value of the positive sample to the negative fluorescence intensity value was calculated as the F_P_/F_N_.

Optimizing the amount of labeling of the FMDV monoclonal antibody 1C6. FMDV-FITS were prepared by labeling fluorescent beads with different amounts of FMDV monoclonal antibody 1C6, as described above.

**1.2 Optimization of T-line antibody concentration**

6D7 of different concentrations (0.4, 0,6, 0.8 and 1 mg/mL) were encapsulated on the T-line of the NC membrane using XYZ3050 Dispensing platforms. Then we prepared SVV-FITS according to the FITS preparation method. The SVV virus solution was then diluted with PBS to 10^7^ PFU/mL as a positive sample, while PBS was used as a negative control. 75 μL of negative sample and positive sample were added to SVV-FITSs and after reaction for 12 min the fluorescence intensity was detected by Fluorescence Immunoassay Analyze. Each test was performed three times, the fluorescence intensity of the positive and negative samples was recorded and the ratio of the fluorescence intensity value of the positive sample to the negative fluorescence intensity value was calculated as the F_P_/F_N_.

The method of optimizing the concentration of antibody 1C6 in the T-line of FMDV-FITS is consistent with the above.

**1.3 Optimization of fluorescent probe concentration**

Preparation of SVV-FITS using fluorescent probe FB-5G6 of different concentrations (1, 3, ,5, 7 and 9 μg/mL). The SVV virus solution was then diluted with PBS to 10^7^ PFU/mL as a positive sample, while PBS was used as a negative control. 75 μL of negative sample and positive sample were added to SVV-FITSs and after reaction for 12 min the fluorescence intensity was detected by Fluorescence Immunoassay Analyze. Each test was performed three times, the fluorescence intensity of the positive and negative samples was recorded and the ratio of the fluorescence intensity value of the positive sample to the negative fluorescence intensity value was calculated as the F_P_/F_N_

The FMDV fluorescent probe FB-1C6 concentration was optimized as described above.

**1.4 Optimization of detection time**

In order to investigate the optimal detection time, the fluorescence intensity on the T-line was recorded at 2 min intervals using a fluorescence immunoassay analyzer after dropwise addition of samples into the sample wells of FITS, and the experiment was repeated three times.

**Results**

**1 Preparation, purification and indirect immunofluorescence analysis of the corresponding antibodies**

**1.1 Determination of ELISA titer of hybridoma cell supernatant**

**
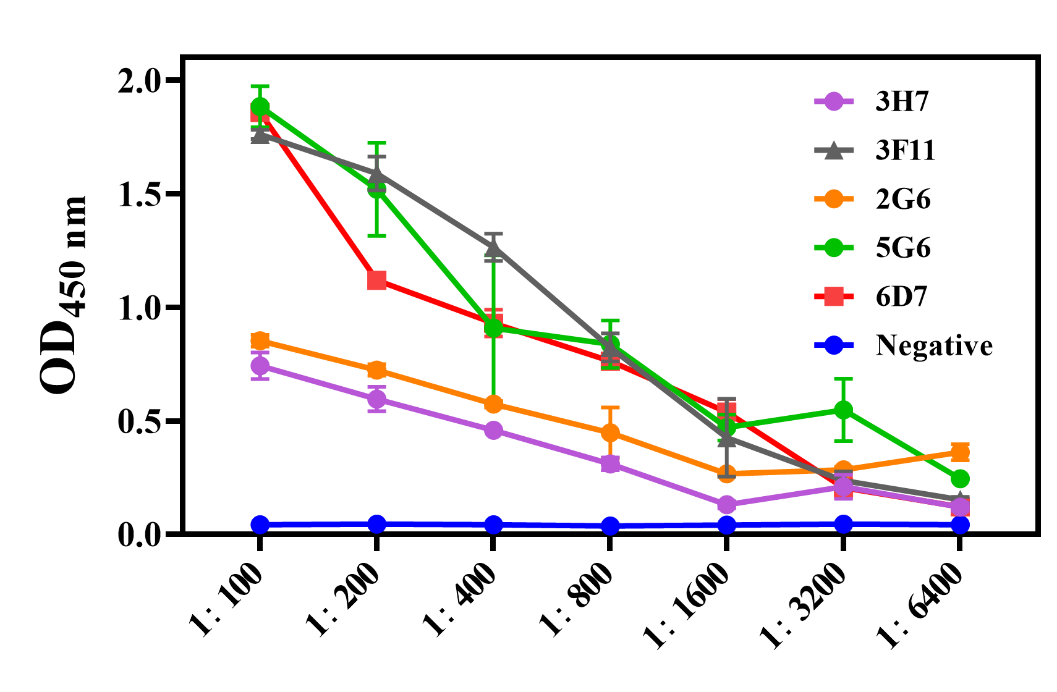
**After cell fusion, we collected supernatants from positive hybridoma cells for ELISA assay. The results were shown in Figure.S1, and a high ELISA potency was observed for 6D7, 5G6 and 3F11 hybridoma cells. Therefore, these three strains of hybridoma cells were selected for ascites preparation and antibody purification.

**Figure.S1.** Determination of ELISA titer of hybridoma cell supernatant

**1.2 Detection of antibody characteristics**

Using Protein A/G column purification of antibodies from the ascites, the purified by SDS-PAGE of ascites were identified. The results showed that the heavy chain and light chain bands could be clearly seen in the purified ascites, and there was no obvious stray band, which indicated that the purity of 6D7, 3F11 and 5G6 antibodies obtained by this method was high (Figure.S2).


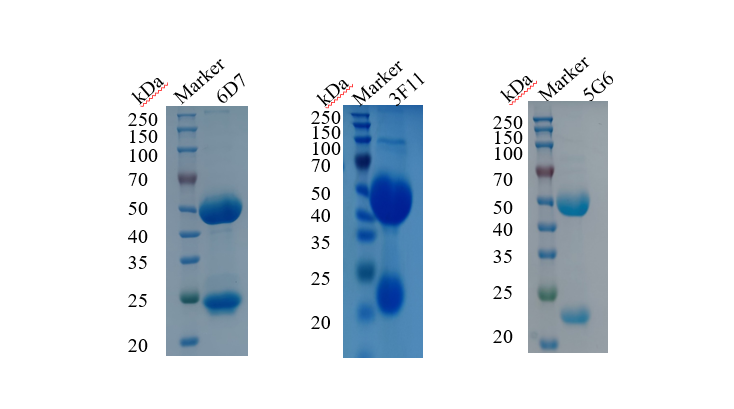
**Figure.S2.** The purified antibody was detected by SDS-PAGE


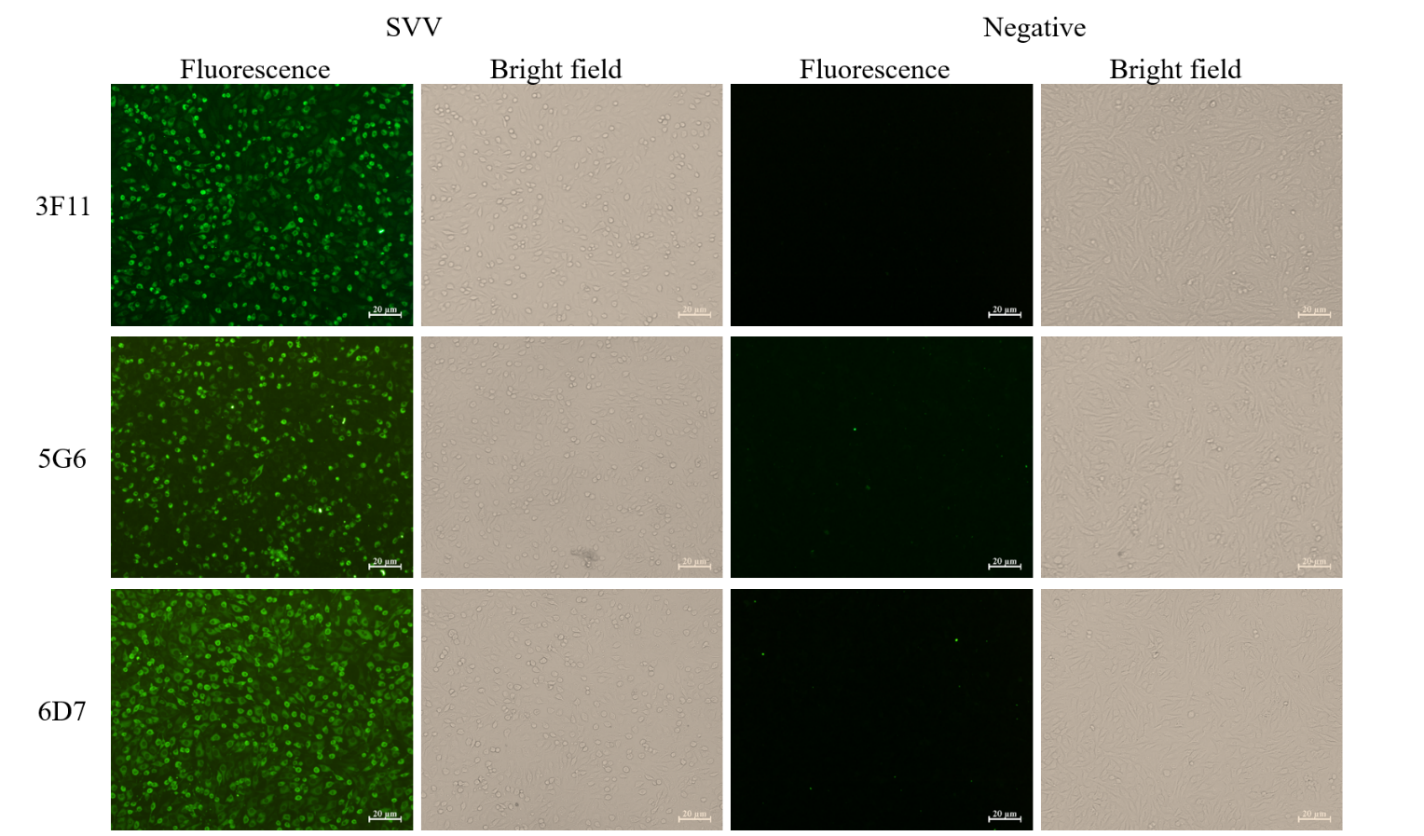
To further determine the antigenic specificity of the 6D7,5G6 and 3F11 antibodies, we verified them by IFA assay. As shown in Figure.S3, mAb 6D7, 5G6 and 3F11 could react with SVV-infected BHK-21 cells, and no fluorescence was detected in mock-infected cells, indicating that mAb 6D7,5G6 and 3F11 could specifically recognize the viral antigens in SVV-infected BHK-21 cells.

**Figure.S3.** IFA was performed 12 h after SVV infected BHK-21 cells. mAb 3F11, 5G6 and 6D7 were used to stain the cells with primary antibodies, respectively.

**1.3 Selection of antibodies pairs**

The optimal detection of the antibody pairs was determined by double-antibody sandwich ELISA after HRPylation of 5G6, 6D7, and 3F11, respectively, using the HRP Rapid Labeling Kit. The results showed the greatest difference in OD_450_ between positive and negative samples when 5G6 and 6D7 were paired for detection (Figure.S4).

**
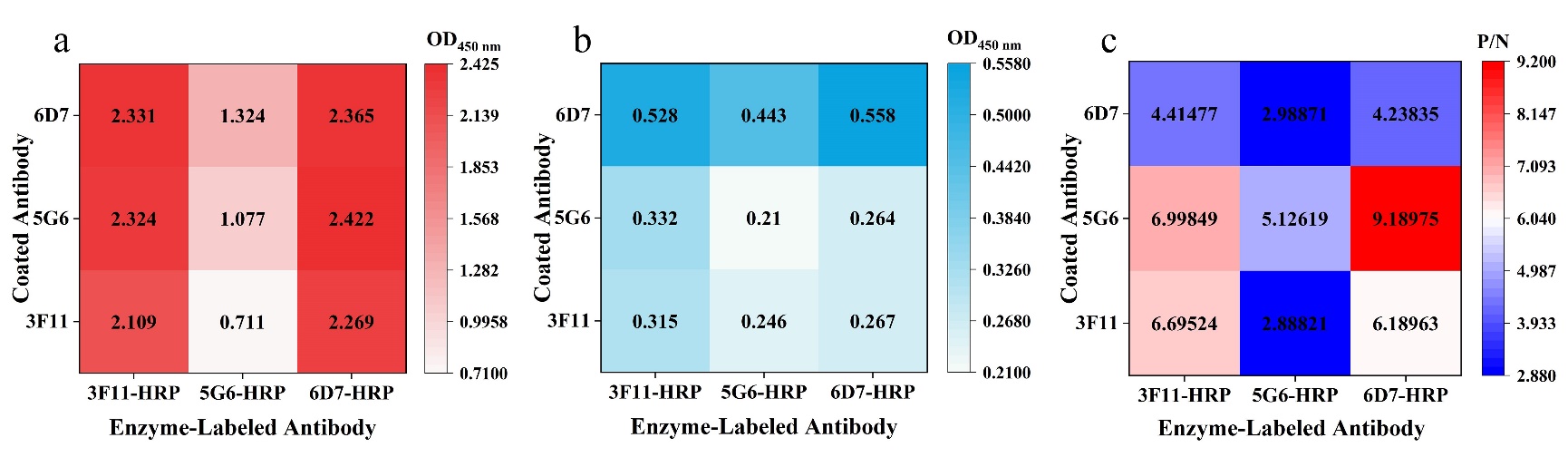
Figure.S4.** Detection of antibody pairs by sandwich ELISA. **(a)** Sandwich ELISA for OD_450_ of positive samples. **(b)** Sandwich ELISA for OD_450_ of Negative samples. **(c)** Ratio of OD_450_ of positive samples to OD_450_ of negative samples

Meanwhile, we prepared three different fluorescent probes (FB-3F11, FB-5G6 and FB-6D7) using 3F11, 5G6 and 6D7 coupled with fluorescence beads, respectively. The Dispensing platforms was then used to encapsulate 3F11, 6D7, and 5G6 on three NC membranes as test-lines. Nine fluorescence immunochromatographic test strips were prepared by combining three fluorescent probes and three NC membranes in different combinations, and the detection effect of the nine test strips was tested by using different titers of viral fluids. As shown in Figure.S5, Fluorescence immunochromatographic test strips prepared with 5G6-labeled fluorescence beads and 6D7 as the test-line were able to maintain the fluorescence intensity at a high level when detecting viral samples while the fluorescence intensity at a low level when detecting the negative samples. This result again shows that the best detection was achieved with the combination of antibodies 5G6 and 6D7.

**
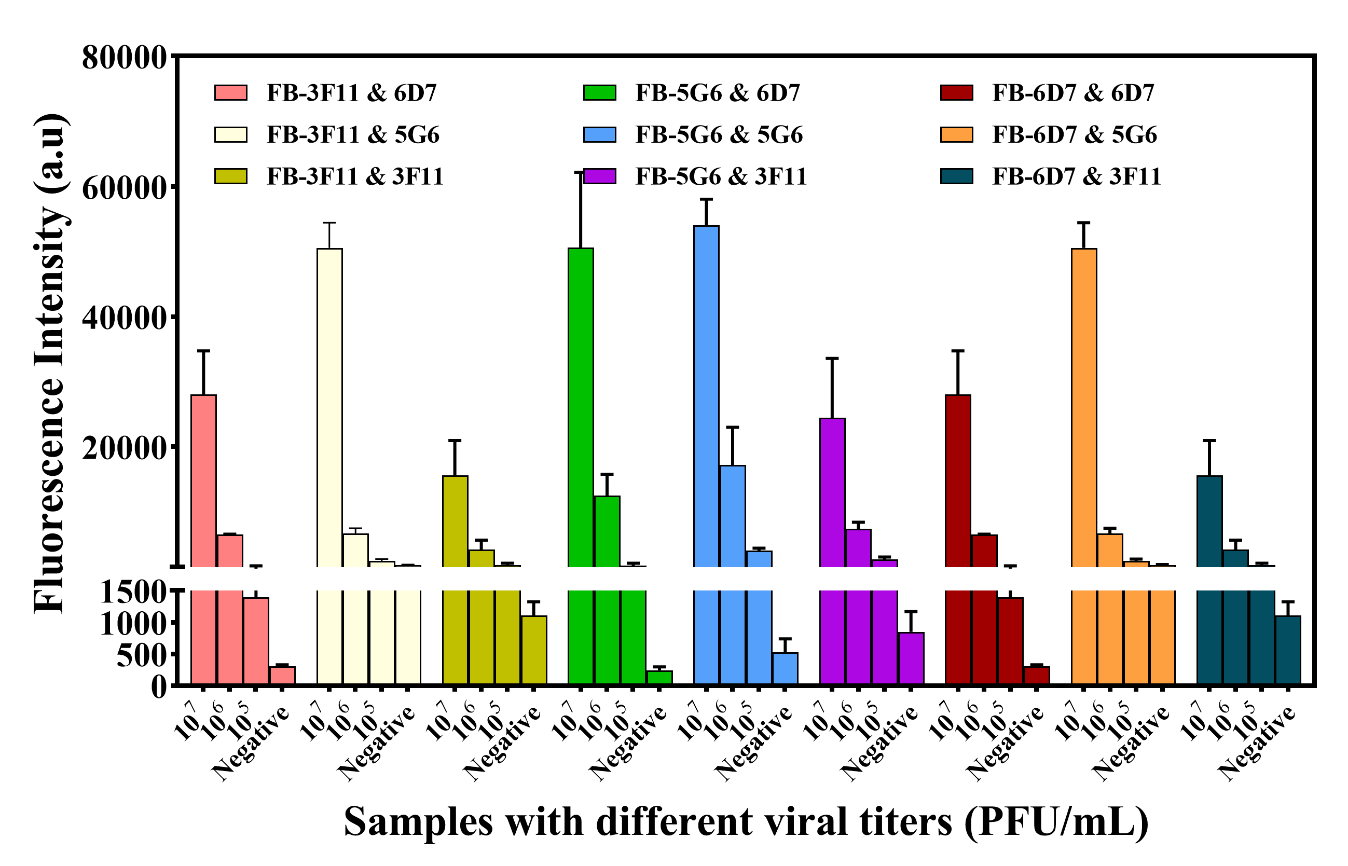
Figure.S5.** Detection of antibody pairs by sandwich FITS.

**2 Optimization of FITS**

**2.1** **Optimization of the amount of antibody labeling**

To further improve the performance of FITS, we optimized the FITS operating conditions. First, we optimized the optimal coupling amount of conjugate between the fluorescent beads and the antibody. We labeled the fluorescence beads with different amounts of antibody and determined the fluorescence intensity using Fluorescence Immunoassay Analyze. We added the prepared positive and negative reference to FITS. After 12 min, the fluorescence intensity was measured using Fluorescence Immunoassay Analyze. The results showed that the T-line fluorescence intensity value reached the maximum when the amount of 5G6 labeled fluorescence beads was 5 μg, The greatest difference in fluorescence intensity, i.e., the greatest F_P_/F_N_ value, was observed between SVV negative and positive samples (Figure.S6a). The highest fluorescence intensity ratio between positive and negative FMDV samples was achieved after labeling the fluorescent beads with 7 μg of 1C6 (Figure.S6b). Therefore, 5 μg of 5G6 labeled fluorescence beads was the best antibody conjugate for detection of SVV, and 7 μg of 1C6 was the best antibody conjugate for detection **
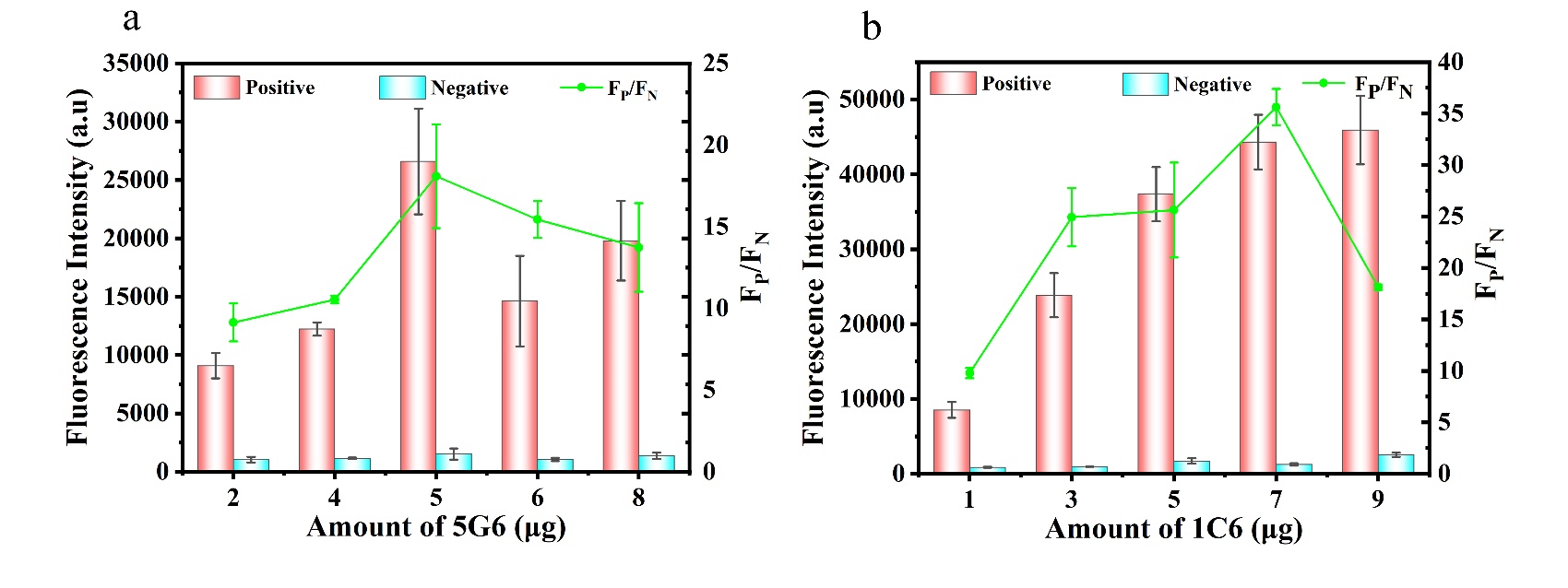
**of FMDV.

**Figure.S6.** Optimization of the amount of antibody labeling. Fluorescence intensity of the T-line at Positive samples and Negative samples by Fluorescence Immunoassay Analyze. F_P_/F_N_ is the ratio of the T-line fluorescence intensity of the test-positive sample to the T-line fluorescence intensity of the test-negative samples. Each value represents the mean of three replicates (n = 3). **(a)** Optimization of the labeling amount of SVV fluorescent probe 5G6 antibody. **(b)** Optimization of the labeling amount of FMDV fluorescent probe 1C6 antibody.

**2.2 Optimization of** **antibody concentration on the T-line**

We then optimized different concentrations of antibodies on the T-line. The results showed in Figure.S7a, which the difference between negative and positive samples of SVV achieved greatest at the concentration of 0.6 mg/mL of 6D7. In Figure.S7b, when the concentration of 1C6 on the T-line was 0.6mg/mL, the F_P_/F_N_ of the tested sample was the largest. Therefore, the optimal concentration of antibody on the T-line was 0.6 mg/mL for detecting SVV and 0.6 mg/mL of 1C6 for detecting FMDV on the T-line.

**
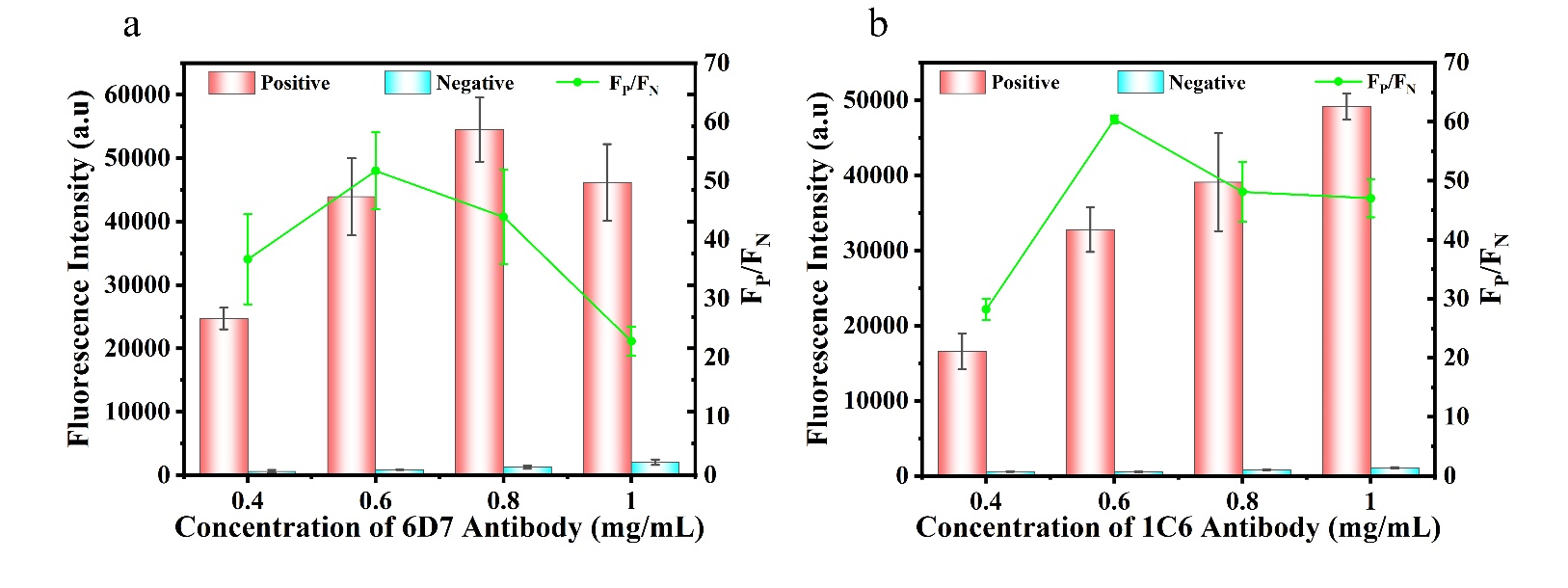
Figure.S7.** Optimization of antibody concentration on the T-line of FITS. **(a)** Optimization of 6D7 concentration on the T-line of SVV-FITS. **(b)** Optimization of 1C6 concentration on the T-line of FMDV-FITS

**2.3 Optimization of fluorescent probe concentration**


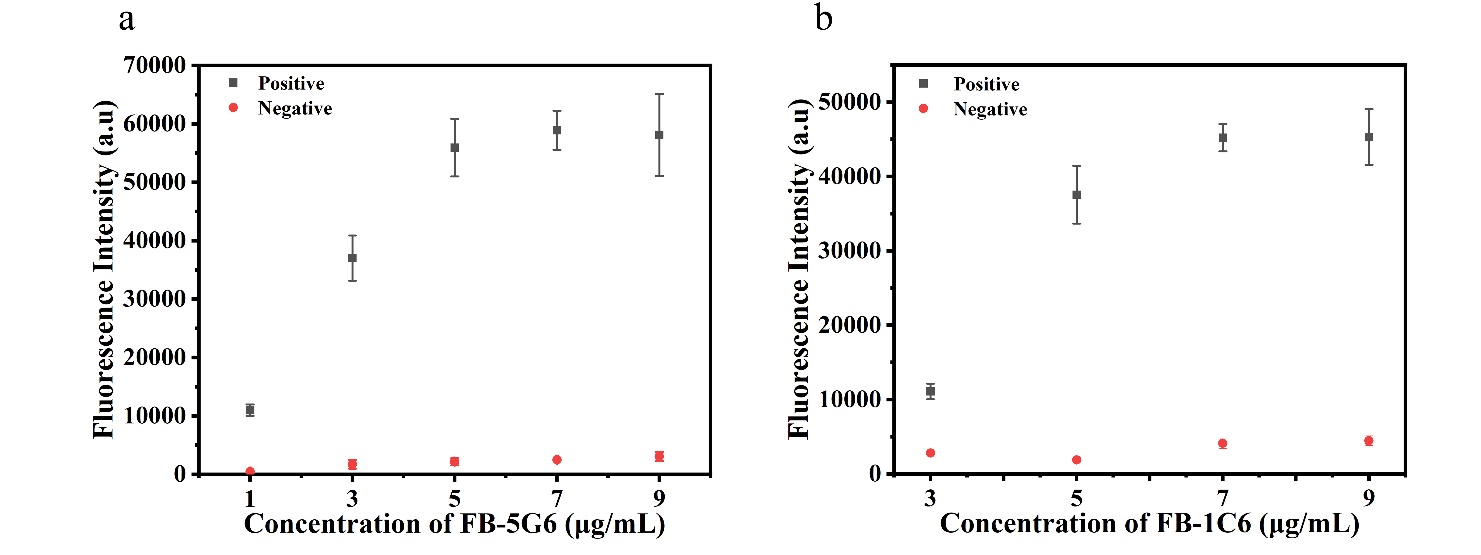
We also explored the effect of fluorescent probe concentration on FITS. The results showed in Figure.S8a and b, when SVV and FMDV were detected, the fluorescence intensity of FITS increased with the increase of the concentration of fluorescent probe, and when the concentration of fluorescent probe exceeded 5 μg/mL, the fluorescence intensity of FITS tended to be flat. SVV-FITS and FMDV-FITS the fluorescent probe (FB-5G6 and FB-1C6) optimal concentration of content are 5 μg/mL.

**Figure.S8.** Optimization of fluorescent probe concentration. **(a)** Optimization of SVV fluorescent probe FB-5G6 concentration. **(b)** Optimization of FMDV fluorescent probe FB-1C6 concentration.

**2.4 Optimization of reaction time of FITS**


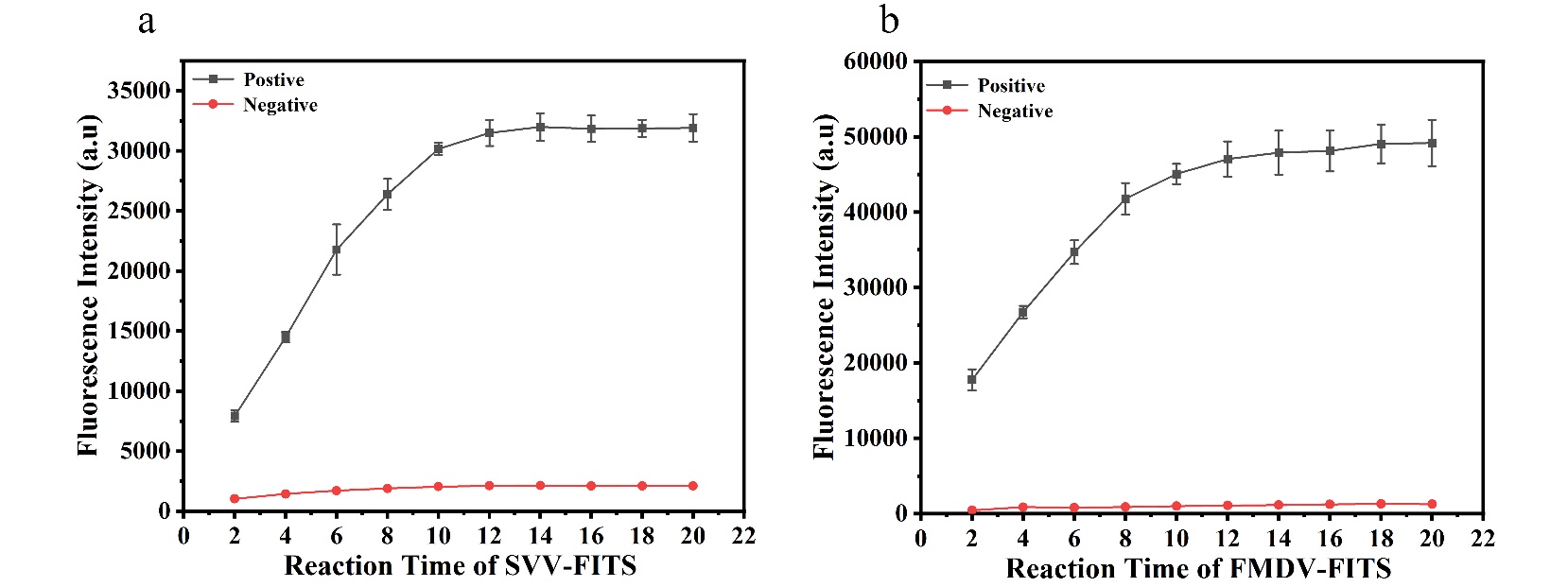
Finally, we also explored the optimal reaction time of FITS, and recorded the T-line fluorescence intensity every 2 min after dropping the sample to FITS. Results show in Figure.S9a and b, the T-line fluorescence signal intensity of SVV-FITS and FMDV-FITS increases with the extension of reaction time, the T-line fluorescence intensity tends to be stable after reacting 12 min. Therefore, 12 min was selected as the best reaction time for FITS.

**Figure.S9.** Optimization of reaction time of FITS. **(a)** Optimization of SVV-FITS detection time. **(b)** Optimization of FMDV-FITS detection time.

**3** **Sensitivity of analysis** **FITS**

We extracted viral RNA from SVV viral fluids of different viral titers and performed conventional RT-PCR. In order to ensure that the amount of virus detected was consistent, we each took 300 μL of virus solution with different viral titers to extract RNA with different viral titers using the Trizol method. And the RNA was reversely transcribed into cDNA using High Efficiency Reverse Transcription Kit purchased from YEASE Biotechnology Company (Shanghai, China). We obtained 20μL of cDNA after reversely transcribed. Then we took 5 μL of cDNA for PCR amplification using specific primers. Finally, the results were analyzed using 1% agarose gel electrophoresis. The results are shown in Figure.S10, with the increase of dilution, the brightness of SVV specific target band gradually decreased. At a viral titer of 10^5^ PFU/mL, the SVV-specific destination band was very dark and close to disappearing. It indicates that the limit of detection of SVV by RT-PCR routinely performed in the laboratory is 10^5^ PFU/mL, which is similar from the limit of
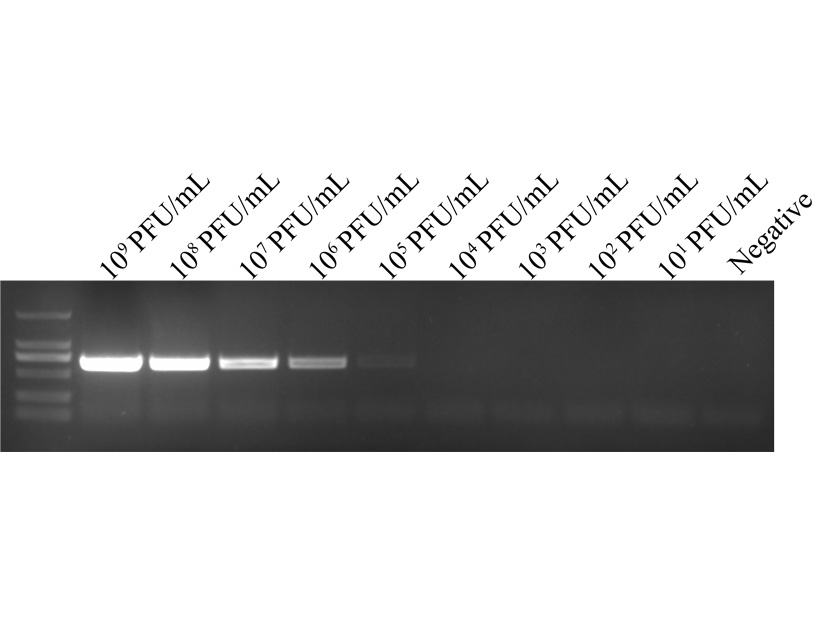
detection of the SVV-FITS.

**Figure.S10.** Results of conventional RT-PCR for SVV.


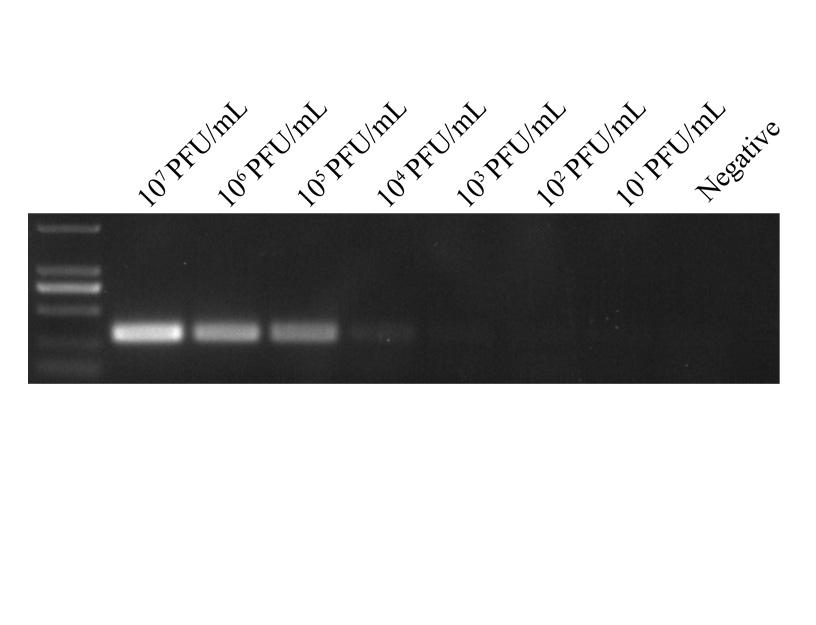
Using the same method we obtained cDNAs for different viral titers of FMDV. We utilized FMDV-specific primers for the cDNA of FMDV to PCR. The results are shown in Figure.S11, a gradually blurred FMDV-specific band can be seen at approximately 450 bp. When the virus titer was 10^4^ PFU/mL virus titer, the band of FMDV nearly disappeared. The detection limit of RT-PCR is 10^4^ PFU/mL. This detection limit is similar FMDV-FITS. However, compared with FMDV-FITS, conventional RT-PCR is more time-consuming.

**Figure.S11.** Results of conventional RT-PCR for FMDV.
